# The Differential Impact of Retirement on Contact Frequency With Family, Friends, Neighbors, and Coworkers

**DOI:** 10.1093/geronb/gbaf042

**Published:** 2025-03-06

**Authors:** Jasper J A Bosma, Kène Henkens, Hanna van Solinge

**Affiliations:** Netherlands Interdisciplinary Demographic Institute-KNAW/University of Groningen and the University Medical Centre Groningen, Groningen, the Netherlands; Netherlands Interdisciplinary Demographic Institute-KNAW/University of Groningen and the University Medical Centre Groningen, Groningen, the Netherlands; Department of Sociology, University of Amsterdam, Amsterdam, the Netherlands; Netherlands Interdisciplinary Demographic Institute-KNAW/University of Groningen and the University Medical Centre Groningen, Groningen, the Netherlands; (Social Sciences Section)

**Keywords:** Gender, Life course, Longitudinal study, Social connectedness, Well-being

## Abstract

**Objectives:**

Most studies on retirement and social network dynamics focus on the closer social network, leaving the role of more peripheral contacts largely overlooked. This article studies how retirement affects contact frequency with a wider range of social ties. We formulate and test differential hypotheses for each category of ties, and additionally examine gender and partner status differences.

**Methods:**

We analyze 3 waves of panel data of the NIDI Pension Panel Study, collected in the Netherlands between 2015 and 2023 (*n* = 5,238). We use 2-way fixed-effects regression models to study within-person change in contact frequency with the different categories of social ties after retirement. To test the differential hypotheses, we conduct Wald tests comparing coefficients across models.

**Results:**

The results indicate that retirement stimulates contact primarily with neighbors and friends, to a lesser extent with siblings and (grand)children, and does not affect contact frequency with parents. Contact with ex-coworkers initially increases but then decreases over time. For women, the positive association between retirement and contact with ex-coworkers, friends, and children is stronger. Not having a partner reduces the association between retirement and contact with friends and ex-coworkers.

**Discussion:**

Our findings suggest that retirees attempt to replace lost workplace interactions by engaging with their former colleagues outside of work and increasing contact with neighbors. Simultaneously, the results suggest substantial continuity in contact with all ties. We suggest that larger changes might take place outside of the ties studied here, with new contacts, and provide several suggestions for future research.

Retirement is increasingly perceived as an opportunity for the development of new identities and the fulfillment of unmet aspirations, instead of a period of withdrawal and relief from work (Henkens & Van Solinge, 2021; Moen, 2016). Correspondingly, scholarly attention has turned to the consequences of retirement for the activities that people engage in (e.g., Grünwald et al., 2021; Tunney et al., 2023), and relatedly, their social engagement (e.g., Comi et al., 2022; Kauppi et al., 2021). So far, the literature on social connectedness in retirement has focused mostly on the structure of the intimate social network. The current study expands the scope of the literature by studying changes in contact frequency with a broader range of social ties, also capturing more peripheral contacts.

Literature on the association between retirement and social connectedness predominately relies on the analysis of discussion networks—the small number (e.g., seven or ten) of contacts with whom respondents discuss personal matters (Comi et al., 2022; Cozijnsen et al., 2010; Kauppi et al., 2021; Patacchini & Engelhardt, 2016; Van Tilburg, 2003). Such approaches have yielded valuable insights into the association between retirement and intimate social network characteristics, such as size and composition. Although core network size tends to be fairly stable, network composition has been found to shift toward emotionally close contacts. Especially work-related ties are likely to be cut in favor of close family (Comi et al., 2022; Kauppi et al., 2021; Van Tilburg, 2003). By design, these approaches do not capture casual, day-to-day interactions with more peripheral ties at work or in the neighborhood. However, workplace social contact is often dominated by such interactions, rather than being a source of emotionally close ties (Dahlin et al., 2008). Therefore, retirement probably constitutes primarily a loss of such casual daily social contact rather than the loss of close ties. There are two reasons to argue that such casual interactions do remain important in retirement. First, frequent interactions with peripheral social contacts contribute to feelings of belonging and can combat loneliness irrespective of the closer social network, rendering them inherently important (Fingerman, 2009; Sandstrom & Dunn, 2014). Second, a diverse network containing various types of social ties can provide access to fulfilling new opportunities and activities (Gee et al., 2017; Granovetter, 1973) and is conducive to maintaining existing and building new social group memberships during life transitions (Haslam et al., 2019). As retirees are increasingly challenged to shape retirement trajectories themselves (Moen, 2016), various types of social contacts can therefore aid adjustment to retirement. Expanding the focus of study toward a broader range of contacts, beyond the core social network, is thus warranted.

A smaller number of studies on the relationship between retirement and social connectedness include a measure of contact frequency. Szinovacz and Davey (2001), for example, include contact frequency in their study on how retirement affects parent–adult child relationships in the United States and found some support for increased contact. Comi et al. (2022), studying discussion networks in Europe, aggregated contact frequency and geographical proximity with individual network members to the social network as a whole, as a proxy for engagement in relationship maintenance. They report no effect of retirement. Likewise, Lim-soh and Lee (2023) include a survey item on contact frequency with friends, coworkers, and relatives as a proxy for informal social participation in South Korea, and find that retirees tend to withdraw from social life. However, focusing on aggregate changes (Comi et al., 2022; Lim-Soh & Lee, 2023) or specific relationships (Szinovacz & Davey, 2001) does not provide a comprehensive view on how contact frequencies with different social network members change upon retirement. Changes in the relative importance of different ties after retirement thus remain obscured.

This study aims to contribute to the literature in three ways. First, this study examines the association between retirement and contact frequency with a comprehensive range of categories of social ties: friends, children, grandchildren, parents, siblings, (ex-)coworkers and neighbors. In contrast to most of the literature, this approach potentially captures a larger and more diverse share of the social network, including day-to-day interactions with peripheral ties. Second, we develop and test hypotheses about the differential impact of retirement on contact with different ties. Our approach thus captures changes in the relative importance of different social ties after retirement, of which contact frequency is indicative (Van Tilburg, 2003). Third, our approach recognizes that the nature of retirement has changed. Although retirement ages have increased, remaining healthy life expectancy around the age of retirement is now higher than before (Henkens & Van Solinge, 2021). Retirement is no longer a one-way withdrawal from the workforce and society that can be captured as a single, discrete event (Phillipson, 2019). This renders the study of how people spend their time after they retire increasingly relevant, both for retirees and society at large. Our analysis also reflects this. We include long-term measures of retirement, postretirement employment, and engagement in other activities that may inhibit or stimulate contact with the different categories of ties. We employ three waves of panel data collected in the Netherlands in 2015, 2018, and 2023 on a sample of 5,238 adults aged 60–65 and employed at baseline, approximately half of which was retired by 2018, and all of which was retired by 2023. These data allow us to study within-person change in contact frequency throughout the retirement transition.

## Theoretical Framework

Several theories such as the Convoy model (Antonucci, 2001), Socioemotional Selectivity Theory (Carstensen, 1992), and the Differential Investment of Resources model (Huxhold et al., 2022) emphasize the dynamic nature of social relationships across the lifespan and in older age specifically. Although these approaches acknowledge the impact of life course transitions such as retirement on social relationships, no specific hypotheses can be derived on how retirement affects the breadth of different categories of social ties studied in this paper. Based on the life course perspective of agency under structure (Elder, 1998), Kalmijn (2012) developed a theoretical lens arguing that life events change the social needs and preferences of individuals, as well as the opportunities that facilitate or constrain their ability to satisfy these needs. This approach has proven fruitful for events such as marriage, divorce, widowhood, parenthood (Kalmijn, 2012), and migration (Spaan et al., 2024). We extend this framework to retirement, which we perceive primarily as a major role transition that individuals cope with in different ways (Bordia et al., 2020).

Employing this perspective, we present four mechanisms through which we expect retirement to affect contact frequency with the various categories of social ties—two related to changes in the opportunity structure and two related to changes in needs. Based on these mechanisms, we formulate hypotheses for each category of ties. Because the meaning and significance of both work and the social network are structured by gender norms (Szinovacz & Deviney, 1999; Umberson et al., 1996), we incorporate gender differences in our theoretical expectations. Finally, because opportunities, constraints, and needs for social contact are likely to depend on whether or not someone has a romantic partner (Dykstra & De Jong-Gierveld, 2004; Kalmijn, 2024), we pose general expectations on the role of partner status.

### Opportunities

Work constrains those engaged in it by structuring and occupying workers’ time and tying them to their workplace (Atchley, 1976). Retirement is likely to alleviate these constraints. Regarding time constraints, work has been found to inhibit frequent contact with close family and friends (Pedersen & Lewis, 2012; Szinovacz & Davey, 2001). Transitioning away from their worker role, retirees may thus become more available to spend time with close ties. However, opportunities for interaction also depend on the availability of those ties. As age peers, friends, and siblings may retire roughly concurrently and thus experience a similar increase in availability. We expect contact with friends to be preferred over siblings due to its voluntary nature (Mares, 1995). Furthermore, we expect this mechanism to be more pronounced among women, who generally engage more in friendship maintenance than men (Oswald et al., 2004), and can thus be expected to spend a larger part of their newly gained time with friends. Likewise, sister-sister sibling pairs have consistently been found to be closer than other sibling combinations (see Stocker et al., 2020), suggesting that women may be more likely to increase contact with siblings than men. Retirees’ parents will also be retired, but their free time is not newly gained, suggesting a smaller change in opportunity for contact. Additionally, because of their advanced age, contact with parents may revolve around the provision of support (Boerner et al., 2022), which has been found to be mostly demand-driven and less dependent on work-related constraints (Grünwald et al., 2021). All in all, we expect that by *reducing time constraints*, retirement is associated with increased interaction—primarily with friends, but also with siblings (especially among women), and to a lesser extent with parents. Partnered people tend to spend at least part of their newly gained time with their partner (Damman et al., 2019), which is why we expect this mechanism to be stronger among unpartnered people.

Upon retirement, people are no longer required to spend time at the workplace, thus reducing place constraints. Consequently, natural opportunities for contact with (ex-)coworkers are lost, as retirees no longer encounter them at work. Instead, retirees tend to spend at least some of their newly gained time in and around their homes (Kalmijn, 2024; Tunney et al., 2023), generating opportunities to encounter neighbors (Seifert & König, 2019). Retirees, therefore, seem likely to engage with their neighbors more frequently. By *reducing place constraints*, we expect retirement to be associated with decreased interaction with (ex-)coworkers, and increased interaction with neighbors.

### Needs

Retirement entails a transition away from the work role identity (Bordia et al., 2020), as well as the loss of casual daily contact at work. We expect retirees to seek to substitute both their work role identity and the daily social interaction they previously engaged in at work. First, unable to continue to rely on their work role identity, retirees may adopt new roles to replace it (Van den Bogaard et al., 2014). One of the most important challenges associated with retirement is the substitution of work-related provisions, for instance, by engaging in activities that provide purpose and structure (Eismann et al., 2019; Moen, 2016). As a “second, deeply gratifying career” (Gauthier, 2002, p. 302), grandparenting is regarded as one of the most rewarding, identity-providing roles available to most retirees (Grünwald et al., 2021). It thus seems likely that upon withdrawal from the worker role, retirees increase contact with their grandchildren and children, as grandparenting will usually involve both. We expect this to be particularly salient among men. Regardless of their employment, women already tend to fulfill multiple social roles before retirement, especially within the family—for instance as grandparent (Chen et al., 2007; Grünwald et al., 2021; Loretto & Vickerstaff, 2013). This is less common among men, for whom an increase in contact frequency with (grand)children thus seems more likely. Particularly among retirees with parents who are still alive, an alternative role would be that of caretaker (Nazroo, 2015), although caretaking—as mentioned before—is often demand-driven. Through the need to *substitute the worker role*, we thus expect retirement to be associated with increased contact with (grand)children (especially among men) and, to a lesser extent, with parents.

Second, transitioning away from the worker role coincides with the loss of casual daily social interaction at work—an important aspect of work that retirees tend to miss greatly (Damman et al., 2015). To compensate for this, retirees may desire more frequent contact with their ex-coworkers outside of work. We expect this association to be more pronounced among women. For men, workplace relations have been found to be mostly functional (Morrison, 2009). This suggests that the significance of these contacts is lost as men transition away from the work role. In contrast, women are more likely to receive social and emotional support from their work-related ties (Morrison, 2009). This support is less tied to the work role and thus more likely to remain valued in retirement, suggesting that women may be more inclined to maintain contact with their ex-coworkers. Furthermore, retirees may seek to substitute workplace interactions with alternatives that resemble them. Accordingly, previous studies indicate that retirees often pursue activities that provide such interactions, like volunteering (Grünwald et al., 2021; Van den Bogaard et al., 2014). Like coworkers, neighbors are ascribed rather than chosen (Völker & Flap, 2007) and may also provide informal, functional interactions resembling those previously provided by coworkers (Wenger, 1990). With increasing age, neighbors often become progressively more important sources of various types of support (Kalmijn, 2024). Retirement could well accentuate this age-related increase in the importance of neighbors in the social network. Combined, we expect that, by fostering a need to *replace daily workplace interactions,* retirement is associated with increased contact with neighbors and, to a lesser extent, with (ex-)coworkers outside of work. We expect the association with coworkers to be especially pronounced among women. Because having a partner provides access to social contact without leaving the house (Eismann et al., 2019), we expect the loss of daily social contact to be greater for unpartnered retirees. In other words, we expect this mechanism to be stronger among the unpartnered.

### Hypotheses

Based on these four mechanisms, we expect positive associations between retirement and contact with each of the seven categories of ties, with variation in strength between the categories. Table 1 summarizes these expectations. We hypothesize that *the association between retirement and contact frequency is the strongest for friends* (hypothesis 1) *and neighbors* (hypothesis 2). Furthermore, we expect that *the association between retirement and contact frequency is moderate for siblings* (hypothesis 3), *children* (hypothesis 4), *and grandchildren* (hypothesis 5). Finally, we expect that *the association between retirement and contact frequency is the weakest for parents* (hypothesis 6) *and (ex-)coworkers outside of work* (hypothesis 7). Regarding gender, we expect that *the association between retirement and contact frequency with friends*, *siblings*, *neighbors*, *and (ex-)coworkers outside of work is stronger for women than for men* (hypothesis 8). Conversely, we expect that *the association between retirement and contact frequency with children*, *grandchildren*, *and parents is weaker for women than for men* (hypothesis 9). We also expect that *the association between retirement and contact frequency with friends*, *siblings*, *parents*, *neighbors*, *and (ex-)coworkers outside of work is stronger for unpartnered retirees* (hypothesis 10).

**Table 1.** Expectations of the Effects of the Different Mechanisms

Mechanism	Neighbors	Friends	Siblings	Children	Grandchildren	Parents	Coworkers
Opportunities							
Time	0	+++	++	0	0	+	0
Place	+++	0	0	0	0	0	—
Needs							
Role loss/substitution	0	0	0	++	++	+	0
Workplace interaction replacement	++	0	0	0	0	0	+
Overall	+++	+++	++	++	++	+	+

Notes: '+' refers to an expected increase in contact frequency as a result of a certain mechanism, while '-' refers to a decrease. '0' refers to no expected changes in contact frequency as a result of the corresponding mechanism.

## Method

### Data

This article employs all three waves of the NIDI Pension Panel Study (NPPS), a large-scale panel study from the Netherlands (Henkens & Van Solinge, 2024). The first wave was conducted in 2015, with follow-ups in 2018 and 2023. Participants were recruited from organizations within the three largest pension funds in the Netherlands. Participants were 60–65 years old at baseline and worked at least 12 hr per week—the threshold for formal labor force participation according to Statistics Netherlands (CBS, 2024). By 2023, all participants had reached the legal retirement age, which gradually shifted from 65 in 2018 to 67 in 2024. The study thus followed the transition into retirement for the complete sample. Of 15,487 questionnaires issued in 2015, 6,793 were completed, constituting a response rate of 44%. For the subsequent two waves, questionnaires were sent to all respondents who participated in the preceding wave. In 2018 and 2023, respectively, 5,312 and 4,261 responses were collected, constituting net response rates of 80.2% and 83.5%. We excluded participants who participated in the first wave only (n = 1,481) and participants who turned out to already have retired before January 1, 2015 (*n* = 74). This reduced our base analytic sample to *n* = 5,238 and (*N* =)14,697 observations.

### Measures

#### Dependent variables

Contact frequency with all categories of ties (friends, children, grandchildren, parents, siblings, (ex-)coworkers outside of work, and neighbors) was measured in each wave by asking respondents how often they see them (in person), on a scale ranging from 1 (rarely or never) to 6 (daily). “Not applicable” constituted a separate answer category, which was treated as missing. The questionnaires included separate questions for contact frequency with youngest and oldest child. In the analyses, we therefore used the average level of contact between the two in case of valid responses to both. Descriptives for the different outcome measures in each wave are summarized in Table 2.

**Table 2.** Descriptive Statistics for All Variables Included in the Analyses

Variable	Year					
	2015		2018		2023	
	Mean/%	*SD*	Mean/%	*SD*	Mean/%	*SD*
*Contact frequency*						
Friends	4.03	0.96	4.12	0.97	4.19	0.96
Siblings	3.32	1.02	3.38	1.06	3.35	1.05
Children	4.46	0.80	4.42	0.81	4.36	0.81
Grandchildren	4.57	0.81	4.62	0.81	4.52	0.81
Parents	4.45	0.90	4.52	0.90	4.53	0.94
(Ex-)coworkers	2.48	1.32	2.67	1.25	2.59	1.17
Neighbors	4.59	1.19	4.69	1.22	4.60	1.17
Retired	3.72%		49.03%		100%	
Time since retirement	0.00	0.00	0.60	0.93	3.94	2.04
Bridge employment	0.00%		6.38%		11.78%	
Comorbidity	1.11	0.86	1.16	0.83	1.17	0.83
Income adequacy	4.06	0.74	4.01	0.75	4.02	0.75
Partner status	0.80	0.40	0.80	0.40	0.78	0.42
Housekeeping	7.14	5.52	7.59	5.72	8.91	6.13
Volunteering	1.14	2.70	1.93	4.21	3.47	5.43
No. of grandchildren 0–5	0.91	1.28	0.98	1.29	0.85	1.20
No. of grandchildren 5–11	0.61	1.16	0.96	1.41	1.30	1.58
No. of grandchildren 12 and above	0.16	0.62	0.37	0.96	0.95	1.53
Moved house	—		6.62%		9.00%	
Fraction of full-time workweek	0.15	0.20				
Gender (female)	45%					

#### Independent variables

We defined retirement as the receipt of pension benefits. In the Netherlands, state pension eligibility coincides with mandatory retirement. Accordingly, respondents aged above the state pension age or who reported receiving pension benefits from an early retirement arrangement were coded as retired. To account for longer-term effects, we also included the time that had passed since retirement. This variable was recoded into categories ranging from 0 (not yet retired/up to 1 year retired) to 8 (more than 8 years). We controlled for postretirement work with a binary variable, bridge employment, indicating whether a respondent worked for pay while receiving pension benefits.

We include various control variables that might be related to retirement and stimulate or inhibit contact with certain ties: comorbidity, perceived income adequacy, housekeeping, volunteering, number of grandchildren in three age categories, and residential relocation. Details on coding and survey questions are included in Supplementary Table S1.

### Analysis

The data were analyzed using two-way fixed-effects (FE) regression models. FE models rely on deviations from within-respondent means only and are thus robust to time-invariant unobserved confounders—but remain sensitive to self-selection or unequal trajectories (Vaisey & Miles, 2017). Two-way FE models additionally include time-dummies to account for contextual changes assumed to affect all respondents homogeneously (Wooldridge, 2021). We included year-dummies to account for the coronavirus disease 2019 (COVID-19) pandemic that occurred between waves 2 and 3.

Because FE models rely on within-respondent changes over time, effects of time-invariant variables, such as gender, can only be estimated through an interaction term, modeling their effect on the association between a time-variant independent variable and the dependent variable (Vaisey & Miles, 2017). For each dependent variable, two models were estimated. The first includes the direct effect of retirement as a key independent variable, whereas the second adds interaction terms of retirement with gender and retirement with partner status at the wave preceding retirement. In these models, we adapted the time-varying partner status variable by splitting it into two separate variables for losing and gaining a partner after retirement. In the data, men worked more hours than women. To isolate the cultural effects of gender as hypothesized in the theoretical framework, the second models also controlled for preretirement working hours. We conducted sensitivity analyses with a three-way interaction of gender, retirement, and partner status. The results of these analyses can be found in Supplementary Tables S2 and S3. Various additional sensitivity analyses, including ordered logit FE models and models using interval-level versions of the dependent variables were also conducted, and are available from the authors upon request. The results of the sensitivity analyses corroborate our substantive conclusions.

Multiple imputation using chained equations with 25 imputed data sets was used to address missing data on the covariates (highest proportion of imputed values: 9.09% for babysitting in 2015; mean proportion of imputed values: 3.53%), but not the dependent variables. Consequently, *N* varies between the models. To account for heteroscedasticity and serial correlation, robust standard errors were applied, which equates to clustering standard errors on the respondent level for FE models.

Because our hypotheses suggest differential effects of retirement on the different categories of social ties, the coefficients of the effect of retirement between the dependent variables were formally compared using Wald tests. This required us to manually demean the variables using one imputed data set and then estimate OLS-regression models. The results (not reported) are near identical to the FE models reported in the results section, with minor differences only appearing from the third digit onwards.

## Results

### Descriptive Results

**Figure 1.**
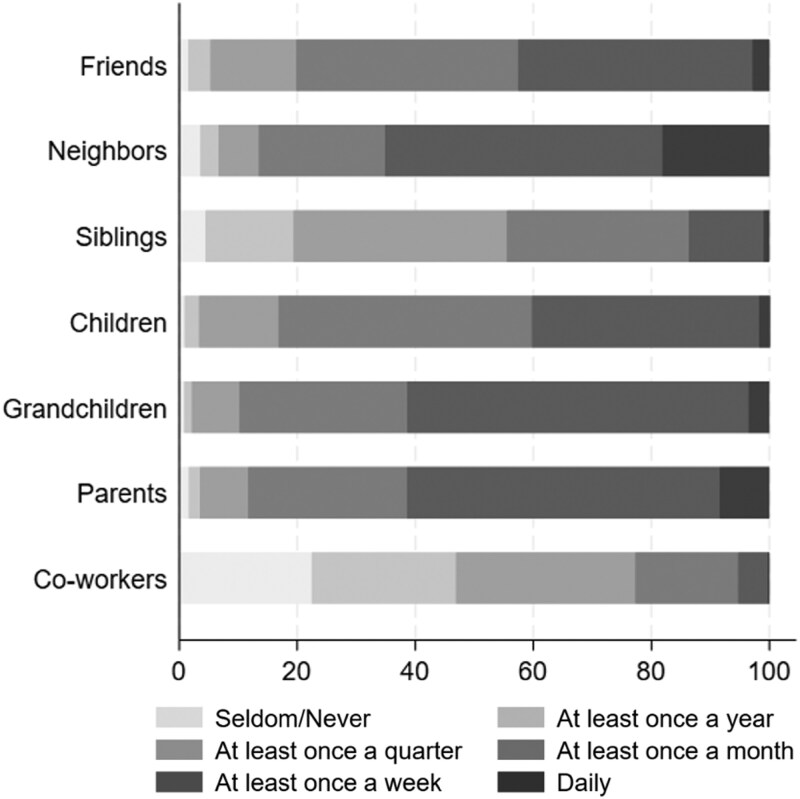
Stacked bar chart of distribution of contact frequency per category of social tie, 2023. As “children” is the calculated mean of two survey-items, it is not restricted to integers. For this figure, any value between 1 and 2 is coded as “seldom/never,” any value between 2 and 3 as “at least once a year,” etc. The original values were used for subsequent analyses.

Figure 1 visualizes the distribution of responses for each category of social ties at the final wave. Respondents reported the highest contact with neighbors, with 65.1% of respondents reporting at least weekly contact. Frequent contact was also reported with parents and grandchildren: 61.4% indicated at least weekly contact with both. Respondents maintained less contact with friends and children: 42.6% and 40.3% reported weekly contact, respectively. Siblings and coworkers were the categories with whom respondents maintained the least contact, with weekly contact reported by 13.6% and 5.3%, respectively.

### The Effect of Retirement on Contact Frequency

Table 3 shows the results of the FE models explaining the association between retirement and contact frequency. Columns 1–7 show the results for each category of ties. First, we will examine the results for each category, where a significant positive association between retirement and contact frequency is a necessary precondition for our hypotheses to be supported. Afterwards, we compare effect sizes between the models to test our hypotheses.

**Table 3.** Fixed-Effects Estimates of Base Models

Variable	Friends	Neighbors	Siblings	Children	Grandchildren	Parents	Coworkers
	*b* (*SE*)	*b* (*SE*)	*b* (*SE*)	*b* (*SE*)	*b* (*SE*)	*b* (*SE*)	*b* (*SE*)
Retirement	**0.139** ^**^ (0.023)	**0.146** ^**^ (0.032)	**0.054** ^*^ (0.023)	**0.059** ^**^ (0.017)	**0.062** ^**^ (0.021)	0.020 (0.041)	**0.115** ^**^ (0.037)
Time retired	0.001 (0.007)	0.008 (0.009)	0.001 (0.007)	0.006 (0.006)	−0.009 (0.008)	0.014 (0.018)	−0.065^**^ (0.011)
Bridge employment	−0.019 (0.032)	−0.039 (0.046)	−0.039 (0.032)	−0.012 (0.025)	−0.019 (0.035)	−0.017 (0.072)	**0.108** ^*^ (0.052)
Comorbidity	0.002 (0.012)	0.002 (0.017)	−0.003 (0.012)	−0.003 (0.009)	0.012 (0.012)	−0.044 (0.023)	0.004 (0.020)
Income adequacy	−0.000 (0.014)	−0.012 (0.019)	−0.006 (0.014)	−0.018 (0.011)	0.012 (0.017)	−0.023 (0.028)	−0.027 (0.022)
Partner status (ref. = unpartnered)	**−0.238** ^**^ (0.052)	−0.127 (0.066)	**−0.177** ^**^ (.046)	**−0.141** ^**^ (0.044)	−0.078 (0.052)	−0.029 (0.101)	−0.144 (0.075)
Hours housekeeping	0.001 (0.002)	**0.004** ^*^ (0.002)	0.003 (0.002)	0.002 (0.001)	−0.001 (0.002)	−0.001 (0.004)	0.003 (0.003)
Hours volunteering	**0.005** ^*^ (0.002)	**0.006** ^*^ (0.003)	0.000 (0.002)	−0.002 (0.002)	0.001 (0.002)	−0.006 (0.009)	0.005 (0.004)
No. of grandchildren 0–5	−0.012 (0.008)	−0.010 (0.011)	−0.003 (0.008)	**0.050** ^**^ (0.006)	**0.052** ^**^ (0.009)	−0.033 (0.020)	0.002 (0.013)
No. of grandchildren 5–11	−0.017 (0.009)	0.008 (0.011)	0.001 (0.009)	**0.032** ^**^ (0.007)	**0.043** ^**^ (0.009)	−0.040 (0.024)	−0.009 (0.014)
No. of grandchildren 12+	0.009 (0.013)	**0.041** ^**^ (0.015)	**0.026** ^*^ (0.011)	**0.031** ^**^ (0.009)	0.021 (0.013)	−0.038 (0.033)	−0.003 (0.019)
Moved house (ref. = no)	0.002 (0.038)	**0.165** ^**^ (0.058)	0.012 (0.037)	−0.051 (0.031)	−0.033 (0.041)	0.060 (0.071)	−0.057 (0.056)
2018	0.027 (0.019)	0.008 (0.025)	0.011 (0.018)	**−0.099** ^**^ (0.014)	−0.001 (0.020)	**0.142** ^**^ (0.030)	**0.141** ^**^ (0.029)
2023	0.005 (0.043)	**−0.238** ^**^ (0.057)	−0.076 (0.042)	**−0.214** ^**^ (0.034)	**−0.116** ^*^ (0.050)	**0.166** ^*^ (0.086)	**0.148** ^*^ (0.070)
Constant	**4.223** ^**^ (0.075)	**4.690** ^**^ (0.096)	**3.473** ^**^ (0.071)	**4.562** ^**^ (0.061)	**4.450** ^**^ (0.086)	**4.660** ^**^ (0.148)	**2.704** ^**^ (0.115)
Obs.	13,802	13,388	12,858	12,296	9,081	3,144	12,608
Ids.	5,178	5,184	4,971	4,527	3,862	1,844	5,130

*Notes*: Values are highlighted in bold when *p* < .05.

^*^ *p* < .05.

^**^ *p* < .01.

Table 3 shows that the impact of retirement on contact with friends, neighbors, siblings, children, grandchildren, and (ex-)coworkers is positive. This increase in contact is particularly salient for friends (*b*(retirement) = 0.139, *p* < .001) and neighbors (*b*(retirement) = 0.146, *p < *.001). Effects were less pronounced for contact with siblings (*b*(retirement) = 0.054, *p = *.018), children (*b*(retirement) = 0.059, *p = *.001), and grandchildren (*b*(retirement) = 0.062, *p = *.004). Column 6 shows no significant association between retirement and contact with parents. Finally, column 7 shows the results for contact with (ex-)coworkers outside of work, which is positively and significantly associated with retirement (*b*(retirement) = 0.115, *p = *.002). However, the effect of retirement on contact with (ex-)coworkers is negated approximately two to three years after retirement, as indicated by the coefficient of time since retired (*b*(time retired) = −0.065, *p < *.001).

The magnitudes of the coefficients for retirement are mostly in line with hypotheses 1 to 5. The results lend no support to hypotheses 6 and 7. We conducted Wald tests to examine statistically whether the coefficients of retirement varied between the models. The results of the Wald tests are reported in Supplementary Table S4. The coefficients for contact with friends and neighbors do not differ significantly from each other but are both significantly higher than those for contact with siblings, children, and grandchildren. In turn, the latter three do not differ significantly from each other. This supports hypotheses 1 to 5, stating that the association between retirement and contact frequency with friends and neighbors is stronger than with siblings, children, and grandchildren.

### Differences by Gender and Partner Status

Table 4 shows the results of the models with interaction terms of retirement with gender and partner status. The main effect can be understood as the effect of retirement for partnered men, while the interactions show how the effect of retirement differs by gender and partner status, expressed as deviation from the main effect. Positive gender interaction effects are identified for contact with friends (column 2) (*b*(retirement × gender) = 0.104, *p = *.001), children (column 5) (*b*(retirement × gender) = 0.066, *p = *.010), and coworkers (column 7) (*b*(retirement × gender) = 0.215, *p < *.001). This suggests that for women, retirement leads to higher increases in contact frequency, with these ties. In the case of coworkers, the main effect loses significance, suggesting that no effect exists for men. The results do not provide consistent support for hypotheses 8 and 9. For partner status, significant interaction effects emerge for friends (*b*(retirement × partner status) = −0.086, *p = *.020), and coworkers (*b*(retirement × partner status) = −0.170, *p = *.003). This suggests that the association between retirement and contact frequency with friends and coworkers is less strong for unpartnered individuals, contrary to hypothesis 10.

**Table 4.** Fixed-Effects Estimates of Models Including Gender and Partner Status Interactions

Variable	Friends	Neighbors	Siblings	Children	Grandchildren	Parents	Coworkers
	*b* (*SE*)	*b* (*SE*)	*b* (*SE*)	*b* (*SE*)	*b* (*SE*)	*b* (*SE*)	*b* (*SE*)
Retirement	**0.119** ^**^ (0.028)	**0.160** ^**^ (0.037)	**0.054** ^*^ (0.027)	**0.040** ^*^ (0.020)	**0.076** ^**^ (0.026)	−0.003 (0.058)	0.076 (0.044)
Retirement × female	**0.104** ^**^ (0.032)	0.080 (0.044)	0.025 (0.032)	**0.066** ^*^ (0.026)	0.016 (0.036)	0.109 (0.067)	**0.215** ^**^ (0.051)
Retirement × unpartnered	**−0.086** ^*^ (0.037)	−0.062 (0.050)	0.050 (0.036)	−0.014 (0.033)	−0.002 (0.046)	0.018 (0.069)	**−0.170** ^**^ (0.058)
Time retired	0.002 (0.007)	0.010 (0.009)	0.002 (0.007)	0.007 (0.006)	−0.009 (0.008)	0.014 (0.018)	**−0.063** ^**^ (0.011)
Bridge employment	−0.013 (0.032)	−0.038 (0.046)	−0.038 (0.032)	−0.007 (0.025)	−0.020 (0.035)	−0.014 (0.072)	**0.118** ^*^ (0.052)
Comorbidity	0.001 (0.012)	0.001 (0.017)	−0.003 (0.012)	−0.003 (0.009)	0.012 (0.012)	**−0.047** ^*^ (0.023)	0.002 (0.020)
Income adequacy	−0.004 (0.014)	−0.009 (0.019)	−0.008 (0.014)	−0.016 (0.011)	0.013 (0.017)	−0.020 (0.029)	−0.019 (0.022)
Gaining partner	**−0.148** (0.107)	**−0.271** ^*^ (0.128)	−0.035 (0.085)	**−0.176** ^*^ (0.073)	**−0.227** ^*^ (0.096)	−0.303 (0.237)	−0.064 (0.124)
Losing partner	**0.211** ^**^ (0.078)	0.200 (0.105)	**0.272** ^**^ (0.081)	**0.154** ^*^ (0.062)	0.093 (0.087)	−0.247 (0.182)	0.053 (0.148)
Hours housekeeping	0.001 (0.002)	0.004 (0.002)	0.003 (0.002)	0.002 (0.001)	−0.001 (0.002)	−0.001 (0.004)	0.004 (0.003)
Hours volunteering	**0.005** ^*^ (0.002)	**0.006** ^*^ (0.003)	0.000 (0.002)	−0.002 (0.002)	0.000 (0.002)	0.007 (0.008)	0.005 (0.004)
No. of grandchildren 0–4	−0.015 (0.008)	−0.012 (0.011)	−0.004 (0.008)	**0.049** ^**^ (0.006)	**0.052** ^**^ (0.009)	−0.036 (0.020)	−0.002 (0.013)
No. of grandchildren 5–11	**−0.020** ^*^ (0.009)	0.007 (0.011)	0.001 (0.009)	**0.032** ^**^ (0.067)	**0.043** ^**^ (0.009)	−0.039 (0.024)	−0.014 (0.014)
No. of grandchildren 12+	0.004 (0.013)	**0.040** ^**^ (0.015)	0.027 (0.011)	**0.031** ^**^ (0.009)	0.022 (0.013)	−0.036 (0.032)	−0.010 (0.020)
Moved house (ref. = no)	0.005 (0.038)	**0.168** ^**^ (0.058)	−0.008 (0.038)	−0.049 (0.031)	−0.028 (0.040)	0.068 (0.068)	−0.055 (0.056)
Work hours at baseline	−0.065 (0.080)	−0.213 (0.114)	−0.131 (0.079)	−0.040 (0.062)	−0.103 (0.081)	−0.133 (0.179)	−0.166 (0.120)
2018	0.032 (0.019)	0.011 (0.025)	0.013 (0.018)	**−0.098** ^**^ (0.014)	−0.002 (0.020)	**0.142** ^**^ (0.030)	**0.146** ^**^ (0.029)
2023	0.005 (0.043)	**−0.246** ^**^ (0.057)	**−0.085** ^*^ (0.043)	**−0.220** ^**^ (0.034)	**−0.119** ^*^ (0.050)	**0.174** ^*^ (0.086)	**0.148** ^*^ (0.070)
Constant	**4.024** ^**^ (0.064)	**4.578** ^**^ (0.083)	**3.342** ^**^ (0.060)	**4.436** ^**^ (0.049)	**4.380** ^**^ (0.075)	**4.628** ^**^ (0.133)	**2.566** ^**^ (0.099)
Obs.	13,802	13,388	12,858	12,296	9,081	3,144	12,608
Ids.	5,178	5,184	4,971	4,527	3,862	1,844	5,130

*Notes*: Values are highlighted in bold when *p < *.05.

^*^ *p < *.05.

^**^ *p < *.01.

## Discussion

Retirement is a major life-course transition that affects individuals’ needs and opportunities for social interaction. Employing large-scale panel data that followed Dutch workers throughout the retirement transition, this article studied how retirement affects contact frequency with a comprehensive range of social ties. We hypothesized that retirement would primarily affect contact with neighbors and friends, to a lesser extent with siblings and (grand)children, and the least with parents and (ex-)coworkers. Although not all hypotheses were supported, the results largely align with the expectations theoretically.

The most notable finding pertains to contact with (ex-)coworkers. The results indicate that after retirement, men initially continue to see their ex-coworkers *outside of work* at preretirement levels, while contact increases among women. Contact with ex-coworkers thus initially seems to persist despite the loss of the primary tie that binds: work. This corroborates the observation that many retirees miss daily workplace interactions (Damman et al., 2015). However, over time, as retirees become further distanced from the work role, and ex-coworkers may also move on into retirement or new jobs, contact does decrease. Nevertheless, these findings add nuance to previous studies that conclude that retirement fosters a (sudden) loss of work-related ties (Comi et al., 2022; Cozijnsen et al., 2010; Kauppi et al., 2021; Van Tilburg, 2003).

Contact with neighbors seems to benefit the most from retirement. Other studies already indicated that neighbors become more important with age (Cornwell et al., 2008; Kalmijn, 2024). The present study confirms this and adds theoretical depth to the role of retirement. Not only do retirees gain new opportunities to encounter neighbors as they spend more time around their house (Seifert & König, 2019; Tunney et al., 2023), interactions with neighbors may also serve to replace former workplace interactions (Völker & Flap, 2007; Wenger, 1990).

At first glance, the results suggest that retirement affects contact with family to a highly limited extent. We find only very modest increases in contact with siblings and (grand)children, and none with parents. In the case of (grand)children, this finding is surprising. Previous research using the same data suggests that grandparenting is one of the prime activities that retirees engage in (Grünwald et al., 2021; Tunney et al., 2023). We suspect that although contact frequency may remain stable, time spent with (grand)children may still increase. Weekly dinners, for instance, may evolve into a full day of babysitting grandchildren. Other familial ties may have settled into stability at this stage in life and are therefore relatively unaffected by life-course transitions (Weiss et al., 2022). Siblings, for example, may meet primarily around birthdays and holidays, and this routine is unlikely to change upon retirement. Further, for people in their sixties, contact with parents is likely to revolve around the provision of support, which is demand-driven (Grünwald et al., 2021) and less affected by work-related constraints. For most of our respondents, parents were already deceased around the time of data collection, as indicated by the relatively low number of observations for contact with parents.

We do find relatively strong increases in contact with friends. Although friendships, like family ties, may have also evolved into a stable dynamic, contact with friends revolves less around ritualistic ceremonies such as birthdays and holidays (Weiss et al., 2022). Compared to family, friendships are voluntary and flexible, and thus more likely to expand upon retirement—especially because friends are often age peers, making them likely to retire roughly simultaneously, and experience a similar increase in free time. Furthermore, whereas the actual number of family bonds (aside from grandchildren) is unlikely to change, new friendships can emerge after retirement, particularly as retirees tend to engage in activities where they may encounter new contacts, such as sports or volunteering (Grünwald et al., 2021; Tunney et al., 2023).

Finally, we found minor gender differences in the associations between retirement and contact with friends, (ex-)coworkers, and children, which were stronger among women than men. Regarding friends, the result aligns with our expectation based on the observation that women tend to be more actively engaged in friendship maintenance (Oswald et al., 2004). Similarly, women’s workplace relations may remain more valuable in retirement than men’s, which are mostly functional (Morrison, 2009). We had expected men to be more likely to increase contact with children, as women are often already more involved in the family before retirement. The results suggest the opposite, which may be attributed to a host of different explanations. Szinovacz and Davey (2001) show that the association between retirement and contact with adult children is complex and depends on various factors, including geographical distance and the child’s gender. The results also contradict our expectations regarding partner status. Unpartnered people seem to increase their contact frequency with friends and coworkers less than the partnered. It seems most likely that the unpartnered already spend a substantial amount of time with friends and coworkers before retirement, leaving fewer opportunities for increased contact.

This study has some noteworthy strengths. Employing a framework of opportunities and needs (Kalmijn, 2012), we present differential hypotheses on the effect of retirement on contact frequency with a broad range of social ties. The hypotheses are tested using two-way fixed-effects models on 8-year panel data with thousands of transitions into retirement. Various sensitivity analyses strengthen the confidence in our substantial conclusions. Notably, although we find significant increases in contact frequency in particular with neighbors and friends, the results also highlight considerable stability in contacts. This might suggest that retirees are more preoccupied with substituting work-related provisions, such as structure and casual day-to-day social contact, than reinvigorating existing relationships. Research has shown that substantial changes occur in the activities that retirees engage in (Grünwald et al., 2021; Tunney et al., 2023), which provide structure and purpose and also involve social interaction—especially with weaker ties. It could thus well be that more important changes in social contact happen by extending the social network through engagement in various activities than by increasing contact with established ties.

This final observation highlights three limitations of this study. First, although our measure of contact frequency has a relatively broad scope, only neighbors and (ex-)coworkers are included as categories of more peripheral social contacts, characterized, on average, by lower levels of emotional involvement. By studying a broader range of more peripheral contacts, for instance, those associated with the activities that retirees engage in, future research could reveal whether larger changes indeed occur beyond the social ties studied here. Our approach also provides no insight into which individual ties are sustained and which are gained (or lost). Distinguishing between new and existing ties could show whether increases in contact are driven by existing ties or by expanding the social network.

Second, the ordinal measure of contact frequency with broad, uneven answer categories does not allow for fine-grained inferences on real-world changes in contact frequency. Future research would benefit from more detailed measures, for instance, by asking respondents to estimate how many days per month they meet someone (e.g., Kalmijn, 2024). Third, our measure is also limited to face-to-face contact despite emerging means of digital communication that have become especially prevalent since the COVID-19 pandemic. Future research should include digital communication, which holds opportunities for social connectedness, but might also reinforce existing inequalities (Neves & Vetere, 2019). As a final note on the COVID-19 pandemic, we included year-dummies to control for the potential longer-lasting effects of the pandemic on contact frequency. This is a common approach for controlling for historic events but assumes that these events homogeneously affect the entire sample (Wooldridge, 2021). Future research employing data collected under regular circumstances is warranted.

Despite these limitations, our article makes important contributions. Studies on social network composition tend to find that work-related ties are promptly cut upon retirement and replaced by family ties (Comi et al., 2022; Cozijnsen et al., 2010; Kauppi et al., 2021; Van Tilburg, 2003). In contrast, this study shows that retirees maintain contact with their former colleagues, at least for some years after retirement. Our results also suggest that contact with (ex-)coworkers may be more likely to be replaced by neighbors than close family. Although literature on the old-old had already started to recognize the importance of neighbors (Cornwell, 2011; Kalmijn, 2024), this so far remained a blind spot in the literature on retirement. In contrast, contact with close family seems to remain fairly stable, although previous research suggests that our measure of contact frequency may not fully capture changes in contact intensity with (grand)children (Grünwald et al., 2021). Nevertheless, we conclude that while intimate contacts may take up a larger share of the closer social network after retirement, the strongest increases in face-to-face contact seem to occur outside retirees’ closest social ties.

## Supplementary Material

Supplementary data are available at *The Journals of Gerontology, Series B: Psychological Sciences and Social Sciences* online.

gbaf042_suppl_Supplementary_Materials

## Data Availability

We describe exclusions, manipulations, and measures included in the study. As original work with the data set has not been completed, the data are not publicly available. However, data, analysis code, and research materials are available from the corresponding author upon reasonable request. Data were analyzed using STATA, version 18.0. This study’s design and its analysis were not preregistered.
