# Supplementary material for: The Differential Impact of Retirement on Contact Frequency With Family, Friends, Neighbors, and Coworkers
Source: J Gerontol B Psychol Sci Soc Sci. 2025 Mar 6;80(6):gbaf042. doi: 10.1093/geronb/gbaf042 (PMC12084831; doi:10.1093/geronb/gbaf042)
Supplement: gbaf042_suppl_Supplementary_Materials [file gbaf042_suppl_supplementary_materials.docx]

***The Journals of Gerontology, Series B: Psychological Sciences and Social Sciences* Supplementary Material: Bosma, Henkens, & van Solinge. The differential impact of retirement on contact frequency with family, friends, neighbors, and co-workers**

**Supplementary Table 1**

*Coding and survey questions* *for all variables included in the analyses*

| Variable | Coding and properties | Item wording |
| --- | --- | --- |
| *Contact frequency* | Ordinal variable coded 1-6  1=rarely or never;  2=at least once a year;  3=at least once a quarter;  4=at least once a month;  5=at least once a week;  6=daily. | ‘How often do you meet the following persons…’ |
| Friends |  |  |
| Siblings |  |  |
| Children |  |  |
| Grandchildren |  |  |
| Parents |  |  |
| (Ex-)co-workers |  |  |
| Neighbors |  |  |
| Retired | Dummy variable, coded 0=working in career job; 1=retired. Respondents that passed the state pension age or left the workforce through an early retirement arrangement were coded as retired. |  |
| Time since retirement | Continuous variable recoded into categories 0-7.  0=less than a year;  7=more than 7 years. |  |
| Bridge employment | Dummy variable coded 0 for non-retired respondents and retired respondents who were not engaged in paid work, and 1 for retired respondents that did engage in paid work. |  |
| Comorbidity | Count variable of 11 different conditions and one ‘other’ option. Any count above 2 recoded to 2. | ‘Do you suffer one or more of the following long-term illnesses, conditions or handicaps (diagnosed by a physician)?’ |
| Income adequacy | Categorical variable ranging from 1 to 5, where 1=very badly, and 5=very well. | ‘How well can you get by with your current income?’ |
| Partner status | Dummy variable coded  0=unpartnered;  1=partnered. | ‘Do you have a husband/wife/partner?’ |
| Housekeeping | Count of the hours spent on housekeeping and volunteering per week. | ‘How many hours do you spend per week on average on the following activities:’ |
| Volunteering |  |  |
| No. of grandchildren 0-5 | Count variable of grandchildren aged below 5 years. Any count above 6 recoded to 6. | For respondents that indicated to have grandchildren: ‘How many per age category?’ |
| No. of grandchildren 5-11 | Count variable of grandchildren aged 5 to 11 years. Any count above 6 recoded to 6. |  |
| No. of grandchildren 12 and above | Count variable of grandchildren aged 12 years and over. Any count above 6 recoded to 6. |  |
| Moved house | Binary variable where 1=moved in the preceding period and 0=did not move in the preceding period. | ‘Which of the following events have you experienced in the previous three/five years (since 2015/2018)?’ |
| Fraction of full-time workweek | The fraction that respondents worked of a full-time (36 hours or more) work week, where 0=full-time and 1=0 hours worked. | ‘How many hours do you work each week?’ |
| Gender (female) | Dummy variable coded 0=male; 1=female. | ‘What is your gender?’ |

**Supplementary Table 2**

*Fixed-effects estimates of models including interaction for gender/partner status combinations*

| Variable | Friends | Neighbors | Siblings | Children | Grandchildren | Parents | Co-workers |
| --- | --- | --- | --- | --- | --- | --- | --- |
|  | b (SE) | b (SE) | b (SE) | b (SE) | b (SE) | b (SE) | b (SE) |
| Retirement (ref.=male, partnered) | **.117**** (.028) | **.157**** (.038) | **.055*** (.028) | **.044*** (.020) | **.084**** (.026) | -.011 (.060) | .083 (.045) |
| *Female, partnered* | **.109**** (.034) | .090 (.047) | .022 (.034) | .052 (.026) | -.014 (.037) | .136 (.074) | **.192**** (.054) |
| *Male, unpartnered* | -.070 (.070) | -.027 (.079) | .042 (.066) | -.081 (.074) | -.165 (.101) | .111 (.130) | **-.246*** (.112) |
| *Female, unpartnered* | .014 (.042) | .010 (.060) | .076 (.039) | .067 (.035) | .046 (.049) | .111 (.089) | .059 (.063) |
| Time retired | .002 (.007) | .010 (.009) | .002 (.007) | .007 (.006) | -.009 (.008) | .014 (.018) | **-.063**** (.011) |
| Bridge employment | -.012 (.032) | -.038 (.046) | -.038 (.032) | -.007 (.025) | -.020 (.035) | -.011 (.072) | **.118*** (.052) |
| Comorbidity | .001 (.012) | .001 (.017) | -.003 (.012) | -.003 (.009) | .012 (.012) | **-.046*** (.023) | .002 (.020) |
| Income adequacy | .004 (.014) | -.009 (.019) | -.008 (.014) | -.016 (.011) | .012 (.017) | -.020 (.029) | -.020 (.022) |
| Gaining partner | -.150 (.106) | **-.277*** (.129) | -.033 (.086) | **-.163*** (.075) | -.193 (.099) | -.340 (.223) | -.051 (.125) |
| Losing partner | **.210**** (.078) | .197 (.106) | **.272**** (.081) | **.158*** (.062) | .101 (.087) | -.247 (.181) | .059 (.148) |
| Hours housekeeping | .001 (.002) | .004 (.002) | .003 (.002) | .002 (.001) | -.001 (.002) | -.001 (.004) | .004 (.003) |
| Hours volunteering | **.005*** (.002) | **.006*** (.003) | .000 (.002) | -.002 (.002) | .000 (.002) | -.007 (.008) | .004 (.004) |
| No. of grandchildren 0-4 | -.015 (.008) | -.012 (.011) | -.004 (.008) | **.050**** (.006) | **.053**** (.009) | -.036 (.020) | -.001 (.013) |
| No. of grandchildren 5-11 | **-.020*** (.009) | .007 (.011) | .001 (.009) | **.032**** (.007) | **.044**** (.009) | -.040 (.024) | -.014 (.014) |
| No. of grandchildren 12+ | .004 (.013) | **.040**** (.015) | .027 (.011) | **.031**** (.009) | .022 (.013) | -.036 (.032) | -.010 (.020) |
| Moved house (ref.=no) | .005 (.038) | **.168**** (.058) | .008 (.038) | -.049 (.031) | -.026 (.040) | .067 (.068) | -.056 (.056) |
| Work hours at baseline | -.068 (.080) | -.220 (.114) | -.130 (.079) | -.030 (.062) | -.084 (.082) | -.153 (.180) | -.150 (.121) |
| 2018 | .032 (.019) | .011 (.025) | .013 (.018) | **-.098**** (.014) | -.001 (.020) | **.142**** (.030) | **.147**** (.029) |
| 2023 | .005 (.043) | **-.245**** (.057) | -.085 (.043) | **-.221**** (.034) | **-.122*** (.050) | .167 (.088) | **.148*** (.070) |
| Constant | **4.023**** (.064) | **4.577**** (.083) | **3.342**** (.060) | **4.436**** (.049) | **4.382**** (.075) | **4.628**** (.133) | **2.569**** (.099) |
| Obs. | 13,802 | 13,388 | 12,858 | 12,296 | 9,081 | 3,144 | 12,608 |
| Ids. | 5,178 | 5,184 | 4,971 | 4,527 | 3,862 | 1,844 | 5,130 |

Notes: Values are highlighted in bold when *p <* .05.

**p* < .05; ** *p* <.01.

**Supplementary Table 3**

*Fixed-effects estimates of models including gender interaction, with different reference categories*

| Variable | Friends | Neighbors | Siblings | Children | Grandchildren | Parents | Co-workers |
| --- | --- | --- | --- | --- | --- | --- | --- |
|  | b (SE) | b (SE) | b (SE) | b (SE) | b (SE) | b (SE) | b (SE) |
| A - Retirement (ref.=male, partnered) | **.117**** (.028) | **.157**** (.038) | **.055*** (.028) | **.044*** (.020) | **.084**** (.026) | -.011 (.060) | .083 (.045) |
| *Female, partnered* | **.109**** (.034) | .090 (.047) | .022 (.034) | .052 (.026) | -.014 (.037) | .136 (.074) | **.192**** (.054) |
| *Male, unpartnered* | -.070 (.070) | -.027 (.079) | .042 (.066) | -.081 (.074) | -.165 (.101) | .111 (.130) | **-.246*** (.112) |
| *Female, unpartnered* | .014 (.042) | .010 (.060) | .076 (.039) | .067 (.035) | .046 (.049) | .111 (.089) | .059 (.063) |
| B – Retirement (ref.=female, partnered) | **.226**** (.037) | **.247**** (.053) | **.077*** (.038) | **.096**** (.029) | .071 (.040) | .125 (.071) | **.274**** (.059) |
| *Male, partnered* | **-.109**** (.034) | -.090 (.047) | -.022 (.034) | -.052 (.026) | .014 (.037) | -.136 (.074) | **-.192**** (.054) |
| *Male, unpartnered* | **-.179*** (.072) | -.117 (.087) | .020 (.070) | -.133 (.075) | -.152 (.103) | -.025 (.130) | **-.438**** (.116) |
| *Female, unpartnered* | **-.094*** (.043) | -.080 (.062) | .054 (.041) | .015 (.035) | .059 (.049) | -.025 (.081) | **-.132*** (.065) |
| C – Retirement (ref.=male, unpartnered) | .047 (.070) | .130 (.079) | .097 (.066) | -.037 (.073) | -.081 (.099) | .100 (.119) | -.163 (.113) |
| *Male, partnered* | .070 (.070) | .027 (.079) | -.042 (.066) | .081 (.074) | .165 (.101) | -.111 (.130) | **.246*** (.112) |
| *Female, partnered* | **.179*** (.072) | .117 (.087) | -.020 (.070) | .133 (.075) | .152 (.103) | .025 (.130) | **.438**** (.116) |
| *Female, unpartnered* | .085 (.076) | .036 (.092) | .034 (.072) | .148 (.078) | .211 (.108) | -.001 (.138) | **.305*** (.118) |
| D – Retirement (ref.=female, unpartnered) | **.131**** (.044) | **.166**** (.063) | **.131**** (.041) | **.111**** (.036) | **.130**** (.050) | .100 (.080) | **.142*** (.066) |
| *Male, partnered* | -.014 (.042) | -.010 (.060) | -.076 (.039) | -.067 (.035) | -.046 (.049) | -.111 (.089) | -.059 (.063) |
| *Female, partnered* | **.094*** (.043) | .080 (.062) | -.054 (.041) | -.015 (.035) | -.059 (.049) | .025 (.081) | **.132*** (.065) |
| *Male, unpartnered* | -.085 (.076) | -.036 (.092) | -.034 (.072) | -.148 (.078) | -.211 (.108) | .001 (.138) | **-.305*** (.118) |

Notes: Values are highlighted in bold when *p <* .05. The table shows the coefficient for retirement for each of the gender/partner status groups, each time with different group as the reference category.

**p* < .05; ** *p* <.01.

**Supplementary Table 4**

*Results of Wald tests comparing coefficients of retirement between models*

| Category | Friends | Neighbors | Children | Grandchildren |
| --- | --- | --- | --- | --- |
|  | *b* (SE) | *b* (SE) | *b* (SE) | *b* (SE) |
| Neighbors | -.008 (.036) | - | - | - |
| Children | **.081**** (.028) | **.090*** (.036) | - | - |
| Grandchildren | **.078*** (.031) | **.086*** (.037) | -.004 (.023) | - |
| Siblings | **.086**** (.030) | **.094*** (.038) | .005 (.028) | .008 (.030) |

Notes: Values are highlighted in bold when *p <* .05. The table shows the differences between the coefficients of retirement in the different models. For each cell, the coefficient for the model in the row is subtracted from the model in the column. As such, in the cell that corresponds with the models for friends and children, the effect of retirement is higher for contact with friends (column) than children (row).

**p* < .05; ** *p* <.01.
